# Bluetongue virus infection creates light averse *Culicoides* vectors and serious errors in transmission risk estimates

**DOI:** 10.1186/s13071-015-1062-4

**Published:** 2015-09-17

**Authors:** Emily G. McDermott, Christie E. Mayo, Alec C. Gerry, Damien Laudier, N. James MacLachlan, Bradley A. Mullens

**Affiliations:** Department of Entomology, University of California, Riverside, CA 92521 USA; Department of Microbiology, Immunology & Pathology, Colorado State University, Fort Collins, CO 80523 USA; Laudier Histology, New York, NY 10024 USA; Department of Pathology, Microbiology & Immunology, School of Veterinary Medicine, University of California, Davis, CA 95616 USA

**Keywords:** Pathogen manipulation, Arbovirus, Vector surveillance, *Culicoides*, Bluetongue

## Abstract

**Background:**

Pathogen manipulation of host behavior can greatly impact vector-borne disease transmission, but almost no attention has been paid to how it affects disease surveillance. Bluetongue virus (BTV), transmitted by *Culicoides* biting midges, is a serious disease of ruminant livestock that can cause high morbidity and mortality and significant economic losses. Worldwide, the majority of surveillance for *Culicoides* to assess BTV transmission risk is done using UV-light traps. Here we show that field infection rates of BTV are significantly lower in midge vectors collected using traps baited with UV light versus a host cue (CO_2_).

**Methods:**

We collected *Culicoides sonorensis* midges in suction traps baited with CO_2_, UV-light, or CO_2_ + UV on three dairies in southern California to assess differences in the resulting estimated infection rates from these collections. Pools of midges were tested for BTV by qRT-PCR, and maximum likelihood estimates of infection rate were calculated by trap. Infection rate estimates were also calculated by trapping site within a dairy. Colonized *C. sonorensis* were orally infected with BTV, and infection of the structures of the compound eye was examined using structured illumination microscopy.

**Results:**

UV traps failed entirely to detect virus both early and late in the transmission season, and underestimated virus prevalence by as much as 8.5-fold. CO_2_ + UV traps also had significantly lower infection rates than CO_2_-only traps, suggesting that light may repel infected vectors. We found very high virus levels in the eyes of infected midges, possibly causing altered vision or light perception. Collecting location also greatly impacts our perception of virus activity.

**Conclusions:**

Because the majority of global vector surveillance for bluetongue uses only light-trapping, transmission risk estimates based on these collections are likely severely understated. Where national surveillance programs exist, alternatives to light-trapping should be considered. More broadly, disseminated infections of many arboviruses include infections in vectors’ eyes and nervous tissues, and this may be causing unanticipated behavioral effects. Field demonstrations of pathogen-induced changes in vector behavior are quite rare, but should be studied in more systems to accurately predict vector-borne disease transmission.

## Background

Pathogen manipulation of host behavior is fascinating from evolutionary, ecological, physiological, and epidemiological standpoints [1, 2]. The idea that one organism can live within the body of another and control its actions intrigues scientists and the general public alike. However, when human and animal pathogens alter the behavior of their insect vectors, the result can be increased disease burdens. When behavioral alterations are adaptive to pathogen spread, there should be an increase in some aspect of vectorial capacity (i.e. the efficiency of transmission), such as biting rates. Increased probing, possibly enhancing transmission, was first demonstrated in the laboratory with *Aedes triseriatus* (Say) mosquitoes infected with La Crosse virus [3]. Since then, several other laboratory studies have demonstrated infection-associated behavioral changes that may increase vectorial capacity, including increased frequency of re-feeding [4], increased movement [5, 6], and improved mating efficiency, enhancing transovarial transmission [7].

Though less well characterized, changes with no impact on transmission may be important for vector control or vector-borne disease surveillance [2, 8]. Altered vector behavior as a result of infection affects transmission risk estimates, and surveillance and control measures. While laboratory studies are important, field evidence is ultimately required to understand how pathogen-induced behavioral changes relate to disease control and surveillance. Such field studies are rare or lacking, especially with arboviruses.

Biting midges in the genus *Culicoides* transmit many mportant animal viruses, including bluetongue virus (BTV), which causes disease in ruminants (e.g. sheep and cattle) with serious economic and animal health impacts [9]. Globally, most *Culicoides* surveillance for BTV is conducted using UV-light-baited suction traps [10, 11], although alternatives include light traps supplemented by CO_2_ [12, 13], traps with CO_2_ alone [13, 14], or rarely, direct collections from sentinel animals [13]. Mayo et al. [13] showed that BTV field infection rates in *Culicoides sonorensis* (Wirth & Jones)*,* the primary North American BTV vector, were lower in insects collected by suction traps baited with both CO_2_ and UV versus traps baited with CO_2_ alone or collected directly from cattle.

We collected *C. sonorensis* from three dairy farms in southern California using UV, CO_2_, and UV + CO_2_ baited suction traps, and tested them for BTV using qRT-PCR to assess differences in estimated infection rates between trap types. We also orally-infected laboratory colony *C. sonorensis* with a BTV-spiked blood meal, and used structured illumination microscopy (SIM) to look at infection intensity in structures of the compound eye. The present study firmly establishes the light effect on BTV-infected insects, shows major spatial heterogeneity of BTV-infected insect activity, discusses consequences for vector-borne disease surveillance, and provides valuable field evidence for pathogen manipulation of host behavior.

## Methods

### Field data

Three dairies in southern California were chosen for the study based on their large populations of *C. sonorensis* determined by preliminary collections. Dairy D and dairy V were located in the Chino Basin, east of Los Angeles in San Bernardino Co., California, USA (approximately 34.00 N, -117.65 W), and dairy S was located in San Jacinto in Riverside Co., California, USA (33.85 N, -117.02 W). The Chino and San Jacinto dairies were separated by approximately 69.2 km and the two Chino dairies were separated by 2.7 km.

All dairies were confinement dairies (cattle on dirt lots fed concentrates and hay), but represented different examples of southern California dairies. Dairy S was the largest of the three in terms of number of cattle and size (~1500 head on 1.59 km^2^), located in a rural valley area. Dairy S had large fields separating the animals and wastewater ponds by several hundred meters, and there was a considerable distance (about 2 km) separating the dairy from the nearest neighbors. Dairy D was smaller (~900 head on 0.30 km^2^). There were two small open fields at dairy D, but feed stalls and open areas separated the animals and wastewater ponds. There were other dairies immediately adjacent to dairy D on three sides. Wastewater ponds at dairies S and D were sampled to confirm that they did serve as developmental sites for *C. sonorensis* immatures. Dairy V was the smallest of the three dairies, both in area (0.21 km^2^) and number of animals (~200 head). During the course of the study there was no wastewater pond in use at dairy V, but runoff water in pastures and along feed stalls proved to be excellent *C. sonorensis* development sites, and large numbers of larvae could be collected from them at any given time.

At each dairy, three CDC-type suction traps (miniature light trap model 512, J. D. Hock and Co., Gainesville, Florida, USA) were used in each of three separated locations (near animals, wastewater ponds, and in fields), except at dairy V, where only two locations were used (animals and fields). The three traps at each location were positioned in a line, 20 m apart, approximately 1 m above the ground and perpendicular to prevailing east–west winds. The traps were baited with CO_2_ (0.5 kg dry ice), battery-powered UV light (F4T5BL, 4 W black light bulb), or CO_2_ + UV light. One of each trap type was set up at each location. The initial positions of the traps within the location were randomized at the start of the study and rotated each night afterwards, so that each trap type was in each position twice to reduce autocorrelation. Insects were collected into a solution of deionized water plus 0.5 % detergent (Liqui-Nox®), keeping the insects in ideal body condition for identification and especially parity sorting, while still being suitable for virus assays (see below) [13]. Traps were set once a week on each dairy for 6 weeks in September and October, which was anticipated to be peak BTV transmission season [13, 14]. Traps were set approximately 2 h before sunset, and collected no more than 4 h after sunrise.

In the laboratory, insects were immediately transferred into 70 % ethanol and stored at -20 °C until they were sorted. *Culicoides sonorensis* were identified by wing pattern and size [15], but are essentially also the only *Culicoides* species collected on southern California confinement dairies [16]. Parity of females was determined visually using a dissecting microscope by the presence or absence of a burgundy-red pigment in the abdominal cuticle of parous midges [17]. Parous midges were usually pooled in groups of 20, or occasionally fewer, and saved in RNAlater® solution (Ambion®) for later viral analysis. Data on the abundance of *C. sonorensis* collected in each trap type by sex and parity status will be published separately.

### Insect pool processing

Pools of parous females were transferred into microcentrifuge tubes with lysis binding buffer solution (AM8500, Ambion/Life Technologies®) and a 1:1 mixture of 0.5 mm and 0.9-2 mm stainless steel beads, and homogenized using a Bullet Blender© STORM (Next Advance Inc.). Viral RNA was extracted using the MagMAX™-96 Viral RNA isolation kit (AM1836, Ambion®). Negative control samples containing only nuclease-free water (*n* = 3) and positive control samples containing pure BTV (BTV-10 ATCC prototype strain) (*n* = 3) were interspersed randomly on each 96-well plate, and were used as controls. The amount of BTV in each sample was quantified by qRT-PCR, using the SuperScript® III Platinum One-Step qRT-PCR kit (11745-100, Invitrogen™). Pools with Ct values <31 were considered BTV-positive [13].

### Structured illumination microscopy

Bluetongue virus infection of the tissues and structures of the midge compound eye was examined using laboratory reared *C. sonorensis* (Van Ryn colony). Insects were infected with BTV (BTV-17 Tulare, CA strain) orally with an infectious blood meal (*n* = 211). A 1:3 ratio of BTV suspension (10^6.7^ TCID_50_) to defibrinated sheep blood (HemoStat Laboratories, Dixon, CA) was used for the blood meal. After blood ingestion, insects were transferred into cardboard containers, provided with 10 % sucrose solution *ad libitum*, and held at 27 °C. At 10 days post-inoculation (dpi), 10 live blood-fed insects were removed and placed into heavy-gel hand sanitizer (62 % EtOH) to be processed for structured illumination microscopy (SIM). At this time point, all competent insects should have achieved fully disseminated infections [18, 19]. Additionally, 10 insects injected with saline solution and kept as above, and 10 non-blood-fed, nulliparous insects were placed into hand sanitizer, and used as negative controls. Sectioning and imaging of all samples was done at Laudier Histology (New York, NY).

At Laudier Histology, insect heads were removed for processing, and the remainder of the bodies were saved for possible future use. Samples were fixed for 48 h in a zinc-acetate based fixative (optimized for arthropods), providing optimal morphological preservation for fluorescence immunohistochemistry (IHC).

After fixation, samples were dehydrated, cleared and processed to a custom hydrophobic acrylic resin. Thin sections were cut at 1 micron and placed on charged glass slides. For the IHC procedure, resin was removed from thin sections, and sections were blocked for non-specific antibody binding and auto-fluorescence. All sections on slides (from positive and negative samples) were incubated with a ready-to-use, mouse-origin FITC-conjugated BTV antibody (CJ-F-BTV-MAB, Veterinary Medical Research & Development) for 2 h.

For negative controls, both positive and negative sample section slides were incubated with a non-immune mouse serum (in lieu of antibody) and then an anti-mouse-FITC secondary.

Structured illumination microscopy was used for imaging. SIM is a fluorescence microscopy approach that breaks through the 240 nm resolution limit of visible light, generating images based on Moiré fringes combined with image reconstruction in Fourier space [20]. SIM provides X and Y resolutions of 85 nm for the fluorescein isothiocyanate (FITC) fluorophore.

### Data analysis

Chi-square analysis was used to look at the numbers of BTV-positive and negative pools from each trap type. Maximum likelihood estimates of infection rates (number infected per 1000 parous females) were calculated using the Excel Add-In, PooledInfRate version 4.0 [21]. Differences in infection rate by trap and trapping location were analyzed by permutational multivariate analysis of variance using distance matrices (R version 3.2.0, Vegan package) (Table 1). Despite the trapping locations at dairy V not being as separated as they were at the other two dairies, their inclusion in the analysis did not affect the significance levels of either the full model or the pairwise comparisons. For this reason, dairy V samples were included in the analyses of site to increase statistical power.

**Table 1:** Effects of Location, Trap and Location*Trap on Infection Rate

Parameter	Df	Sum of Squares	Mean Squares	F. Model	R^2^	Pr (>F)
Location	2	0.001	0.0007	4.51	0.067	0.011*
Trap	2	0.003	0.001	8.27	0.123	0.001***
Location*Trap	4	0.002	0.0004	2.68	0.080	0.033*
Residuals	98	0.016	0.0002		0.723	
Total	106	0.021		1.00		

Permutations: 999

Significance codes: 0 (***), 0.01 (*)

ANOVA table of final model used for infection rate analysis

Week of collection was considered the level of replication and so was not included as a factor in the model. The interactions between dairy and trap or site were not significant, and so dairy was also not included as a factor in the final model. Effect of trap, site, and the interaction between trap and site were significant (*p* < 0.05), with the effect of trap being the most significant (*p* = 0.001). Pairwise comparisons were used to look at differences in midge infection rates between traps (averaging all sites) or between sites (averaging all traps). Infection rate error was calculated by dividing the mean infection rate for CO_2_ traps on a given week by the mean infection rate for UV traps on that week.

## Results and discussion

Because BTV is not transovarially transmitted [22] only previously blood-fed (parous) females were tested for BTV. Of 674 total pools (representing about 13,000 parous female midges), 212 were from CO_2_ traps, 145 were from UV traps, and 317 were from CO_2_ + UV traps. Of the tested pools, 126 (18.7 %) were positive for BTV (Ct < 31).

**Fig. 1:**
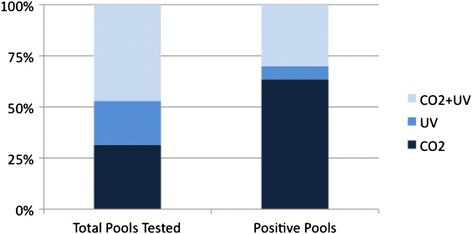
Proportion of BTV-Positive Pools. Proportions of vector pools tested (*n* = 674) for BTV and of the BTV-positive pools (*n* = 126) by trap type

Pool sizes (parous insects per pool) did not differ significantly for the three trap types (*p* = 0.421). When the data from all three dairies were combined, there was an overall highly significant difference between the traps (*×*^2^ = 76.52, df = 2, *p* < 0.001) (Table 2). Despite comprising similar proportions of the total pools, CO_2_ traps and UV traps accounted for vastly different proportions of the positive pools. Infected midges were not well represented in UV-only traps (Fig. 1), a trend maintained across all three dairies. All pairwise comparisons were significantly different (*×*^2^ ≥ 4.65, df = 1, *p* ≤ 0.031), with CO_2_ traps having the highest total number of positive pools (*n* = 80). Relative to UV traps, 10 times as many positive pools came from CO_2_ traps. When the collections from each dairy were examined separately, CO_2_ traps still had higher numbers of positive pools (*×*^2^ ≥ 8.02, df = 1, *p* ≤ 0.005). At two of the dairies (S and V), there was no difference between the UV and CO_2_ + UV traps (*×*^2^ ≤ 1.74, df = 1, *p* ≥ 0.188).

**Table 2:** *×* ^2^ Analysis of Positive vs. Negative Pools by Trap

Trap	Positive pools	Negative pools	Total
CO_2_	80	132	212
	39.6	172.4	
UV	8	137	145
	27.1	117.9	
CO_2_ + UV	38	279	317
	59.3	257.7	
Total	126	548	675

*×* ^2^ = 76.52, df = 2, *p* < 0.001

Within trap type, the first row of the table shows the observed values and the second row shows the expected values

**Fig. 2:**
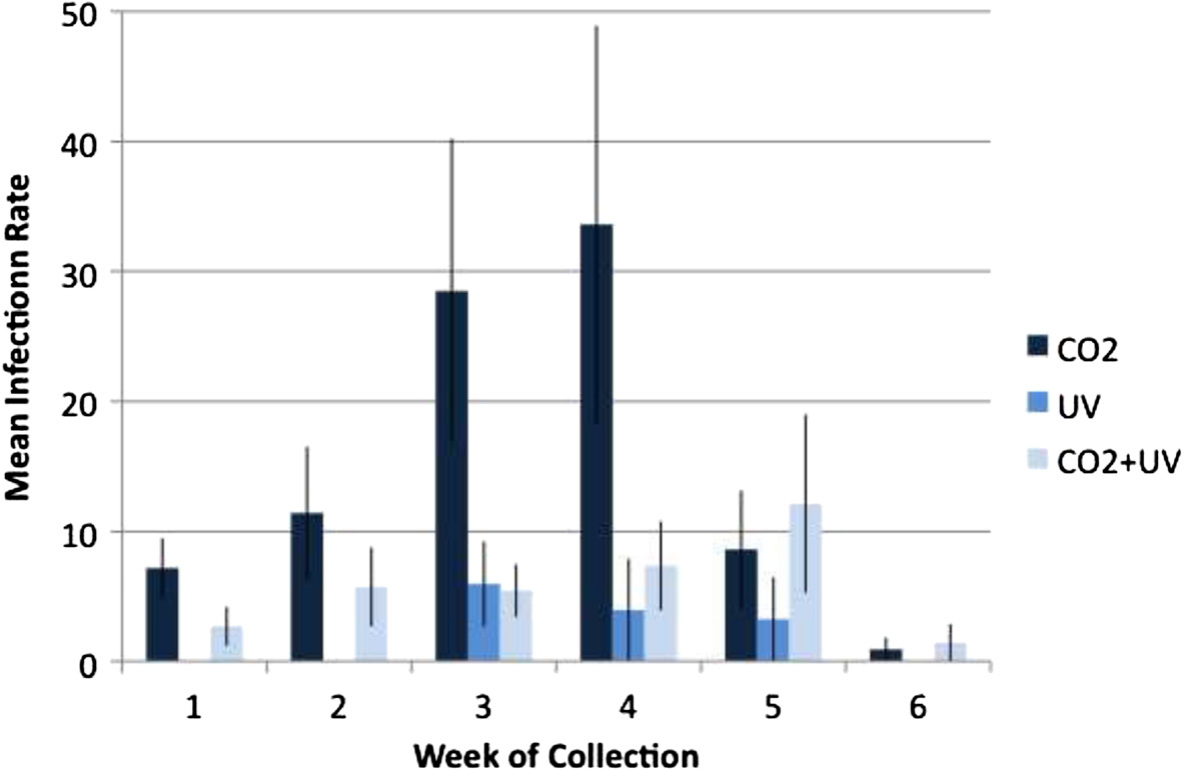
Weekly Infection Rates. Mean (± standard error) infection rate (per 1000 insects) by trap type and week

Across dairies, maximum likelihood estimates of infection rates (reported as number of infected insects per 1000) of UV trap collections (mean = 2/1000) were always markedly lower than those of CO_2_ trap collections (mean = 15/1000) (*p* ≤ 0.021). UV traps only detected BTV when it was most common, and entirely missed detecting BTV on weeks 1, 2 and 6 (Fig. 2). Infection rate estimates of CO_2_ + UV trap collections (mean = 6/1000) were also significantly lower than those of CO_2_ trap collections (*p* = 0.031), suggesting that light actually repelled infected midges. An alternative explanation is that some proportion of uninfected, parous midges are attracted to UV, but not CO_2_ [23]. This could increase the number of total midges collected in the CO_2_ + UV traps, but decrease the observed infection rates in those traps. However, CO_2_ + UV traps had only half as many BTV-positive pools as CO_2_ traps, supporting the pathogen manipulation hypothesis.

Fully disseminated virus infections of insect vectors may include high infection rates in the head and nervous tissues, including the eyes, and infections of these tissues could entail behavioral modification [24–26]. We suspected that BTV also infected *C. sonorensis* eyes during the later stages of infection, and that this may be the root cause of the infection-associated behavior we observed in the field.

**Fig. 3:**
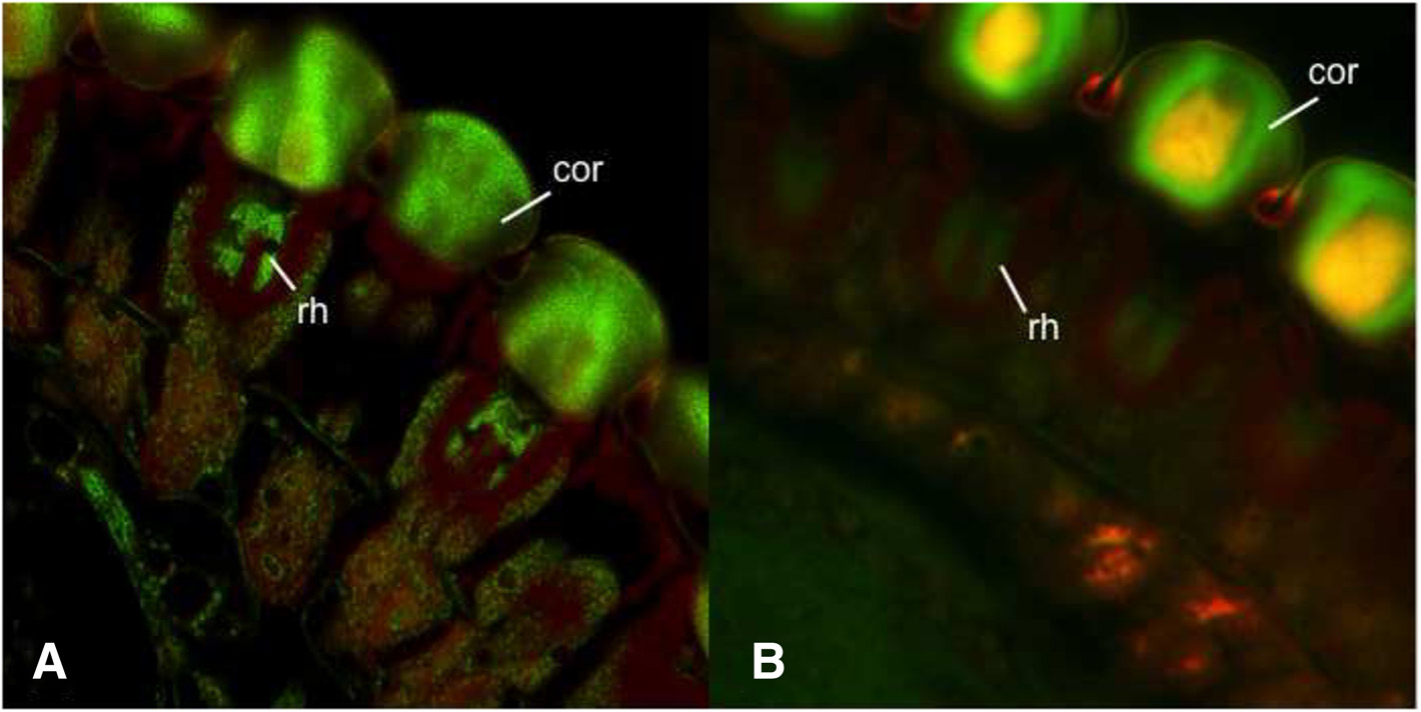
BTV Infection in *Culicoides* Compound Eye. **a** Ommatidia (eye cross-section) of orally BTV infected *C. sonorensis*, after 10 days. **b** Ommatidia of uninfected (un-fed control) *C. sonorensis*. The rhabdom (rh) and cornea (cor) are heavily infected in BTV-positive insects (punctuated green staining)

Ommatidia in the eyes of infected insects showed positive staining, indicating that BTV infected these tissues. The *Culicoides* eye structure closely resembles the dark-adapted arrangement of *Anopheles* mosquito ommatidia [27], and the strongest positive signals were from the cornea and rhabdom (Fig. 3), which collect and focus light to form images [27]. Virus damage in the rhabdom could allow some visible light to essentially be lost, reducing visual acuity. Due to the apparent aversion to light, infection may also be altering how visual signals are processed in the nervous system. Though any adaptive advantage for pathogen spread is unclear, the effect in the field is strong, and it alters our estimates of transmission risk.

For the two dairies (S and D) that had clear separation of trap locations (near animals, near wastewater ponds where midges developed, or in open fields), chi-square analysis was used to examine the number of BTV-positive and negative pools at each location (Table 3). Taken together, there was an overall significant difference among locations (*×*^2^ = 17.36, df = 2, *p* < 0.001). Positive pools were particularly rare from wastewater pond collections, with significantly fewer positive pools from traps in those locations than from traps near both animals and fields (*×*^2^ ≥ 5.53, df = 1, *p* ≤ 0.019), which did not differ from each other (*×*^2^ = 2.06, df = 1, *p* = 0.152). At either dairy separately, there was no difference in the numbers of positive pools from animals versus wastewater ponds locations, although there was a stable trend towards more positive pools in traps near animals (*×*^2^ ≥ 2.59, df = 1, *p* ≥ 0.067).

**Table 3:** *×* ^2^ Analysis of Positive vs. Negative Pools by Location

Location	Positive pools	Negative pools	Total
Animals	14	68	82
	15.6	66.6	
Wastewater Ponds	7	103	110
	27.1	117.9	
Fields	69	211	280
	53.4	226.6	
Total	90	382	472

*×* ^2^ = 17.36, df = 2, *p* < 0.001

Data from dairies S and D only. Within a location, the first row of the table shows the observed values and the second row shows the expected values

**Fig. 4:**
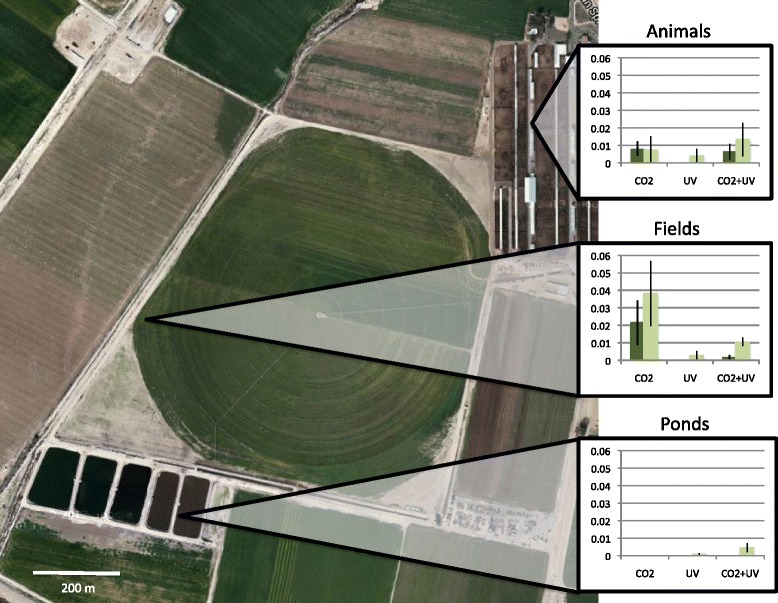
Interaction Between Trap and Location. Mean (± standard error) BTV infection rate (per 1000 insects) by attractant type and trap placement at dairies S and D. Map shows placement of traps on dairy S. Dark green bars represent dairy S, light green bars represent dairy D

The effect of trap placement on infection rate was also examined on dairies S and D (Fig. 4). There was a significant interaction between trap and location (*p* = 0.033), with traps set in fields collecting midges with higher infection rates when baited with CO_2_ alone (mean = 29/1000). Traps placed near wastewater ponds consistently yielded almost no BTV, and when they did collect infected insects, the estimated infection rates from those traps were significantly lower than from traps set either near animals or in fields (*p* ≤ 0.009). UV traps similarly did poorly, regardless of where they were set (mean ≤ 2/1000). There was no difference in the infection rates of midges collected near animals or in fields (*p* = 0.681).

Although geographically separate vector populations may show differences in competence [28], it was surprising to observe substantial differences in infection rates among locations on two individual farms. Distances between trap locations on a farm (200-1000 m) were well within *C. sonorensis* flight range (1-2 km) [29, 30]. We can assume then that the insects belonged to the same interbreeding population, and that infection rate differences among trap locations are unlikely to reflect genetic variation in competence. Further, the same trap location effect appeared on dairies 69 km apart, i.e. in different vector populations.

Detecting higher infection rates in fields and almost no BTV near ponds could reflect an additional virus-mediated behavioral change if infected insects have a longer interval between oviposition and host seeking than uninfected insects. Infected insects may leave oviposition sites (wastewater ponds), ignoring those traps, and disperse further (in this case into fields) before being stimulated by host cues, like CO_2_. Though we did not test this hypothesis, this effect could have major epidemiological consequences, and merits further investigation.

Vector control and disease surveillance programs worldwide rely on a variety of trapping methods to collect insect vectors in order to assess the risk of pathogen transmission to humans and animals. Pathogen manipulation of host behavior has become a popular research topic, but little attention has been paid to how pathogens may change vector behavior in ways that prevent their detection by our surveillance methods. Although these effects may not be as direct as those on transmission, the consequences of ignoring them could be profound, as we have demonstrated for BTV.

Entomological inoculation rates (EIR; infective vector bites per unit time) are field-derived but seldom determined for most vector-borne diseases. Given the many factors that influence vector-host location and biting, collections directly from a host are preferable [31]. However, determining actual biting rates is difficult and especially rare in animal vector-borne disease systems [13, 32], and true EIR determinations directly from animals in the field have not been done for BTV to our knowledge. In the best-studied vector-pathogen system (*Anopheles* mosquitoes and *Plasmodium*), light traps are imperfect, but provide an adequate representation of EIR in some situations [31]. Unlike *Plasmodium*, arboviruses are frequently widely disseminated and capable of infecting many tissues [24–26, 33], with possible repercussions for vector behavior. In both human and animal systems, trap-generated EIR data would be far more efficient logistically and would avoid exposing vertebrates to pathogen transmission, but only provided they represent biting and infection adequately.

Surveillance data, including trap-derived EIR estimates, influence our estimates of vectorial capacity and force of infection. An error in vector infection rates would serve as a direct multiplier of overall error for transmission risk estimates. We calculated the error caused by using only UV trap-collected *C. sonorensi*s, assuming that BTV infection estimates in vectors from CO_2_ traps accurately represent virus in host-seeking insects [13]. When it could be calculated at all, using infection rates from vectors collected in UV traps underestimated those in host-seeking insects (CO_2_ traps) by factors of 2.7–8.5. Additionally, UV traps frequently failed to detect BTV when it was present, particularly early in the transmission season, resulting in errors even higher than could be calculated.

Errors generated by reliance on trapping methods that do not accurately represent infection rates in vectors have serious potential consequences for human and animal health, international trade restrictions, and national economies. For example, the massive European BTV outbreak from 2006 through 2008 cost affected individual countries between 32.4 and 175 million Euros [34, 35]. Estimates from currently BTV-free countries suggest that virus reintroduction would incur similar costs of lost production and control [36]. Using only light traps for surveillance, we could fail to detect BTV activity for weeks or longer, missing critical opportunities for early action to prevent disease spread. Further, UV light traps might not detect BTV during periods when it is not abundant (i.e. potential overwintering in vectors), resulting in misunderstanding of the true activity patterns of BTV in nature.

Biological data from trap collections are also vital for modeling pathogen spread across landscapes (and borders), and for predicting the economic impact of introduction and establishment. Pathogen-associated behavioral changes in vectors should be considered. Using only light trap data may negatively influence predictive models, which inform vaccination campaigns and international trade policy. Surveillance costs should be included in economic impact models, and can be a significant part of total outbreak costs [36–38]. Therefore, optimizing surveillance strategy is critical.

## Conclusions

UV-light baited traps rarely collect BTV infected *C. sonorensis* midges, and in fact, infected midges appear to be UV-light averse. Global reliance on light trapping for BTV vectors may be resulting in transmission risk estimates that are severely understated, and could potentially prevent early detection of BTV outbreaks. Beyond BTV and animal health, light trapping can be used to survey other vector species, including vectors of human pathogens. We have also shown that location of sampling can dramatically impact these estimates, at least for BTV. These data are used by public health officials to time control measures to protect the general public from disease. Little information exists regarding trap influences on estimating infection rates in most other pathogen-vector systems. Accurate prediction of pathogen transmission risk should save human and animal lives, and reduce costs. A better understanding of how pathogens manipulate vector behavior in the field will help improve our surveillance methods and ultimately reduce transmission.
